# Phylogenetical coherence of *Pseudomonas* in unexplored soils of Himalayan region

**DOI:** 10.1007/s13205-016-0493-8

**Published:** 2016-08-13

**Authors:** Stuti Sah, Rajni Singh

**Affiliations:** Amity Institute of Microbial Biotechnology, Amity University, J-3 Block, Sector 125, Noida, UP 201301 India

**Keywords:** *Pseudomonas*, Plant growth promoters, Siderophore, Genetic diversity

## Abstract

*Pseudomonas*, an enormously diverse genus of the γ-*Proteobacteria*, is an important member of soil microbial communities. In this study, genetic heterogeneity and plant growth promotory property of *Pseudomonas* was compared within the group *Pseudomonas* sensu stricto isolated from the lesser explored niches of Himalayan region. A significant difference (*P* < 0.001 in total *Pseudomonas* count) was observed among the six types of soil samples collected from oak forest, fine forest, and agricultural soil. The highest numbers of bacteria were isolated from oak forest soil followed by pine forest soil and agricultural soil. 23.52 % of the total 238 isolates were siderophore producers and were identified as *Pseudomonas* on the basis of PCR amplification using 16S rDNA *Pseudomonas* specific primer. The molecular analysis by Jaccard’s similarity coefficient resulted into eight different clusters and six outlying branches. Some of the clusters include *Pseudomonas* from forest as well as agricultural land. Among the 51 isolates 100 % were siderophore and IAA producers, 68.62 % were phosphate solubilizers and 62.74 % HCN producers. The results reveal that isolates from lesser explored area possess beneficial properties and show genetic heterogeneity among them.

## Introduction

The genus *Pseudomonas* encompasses arguably one of the most complex, diverse, and ecologically significant group of bacteria on the planet. Members of the genus are found in large numbers in all the major natural environments (terrestrial, freshwater, and marine) and also form intimate associations with plants and animals. This universal distribution of *Pseudomonas* suggests a remarkable degree of physiological and genetic adaptability (Spiers et al. 2000). Thus, it is essential to understand the bacterial community structure and diversity in relation to environmental factors and ecosystem functions (Torsvik et al. 1996).

The term Pseudomonad is derived from the Greek word Pseudo (=false) and monad (=a single unit) and literally means “false unit”. The earliest classification of Pseudomonads was based on cultural characteristics, such as rod shape, Gram negative reaction, and motility by means of polar flagella. The members of previously described *Pseudomonas* are now classified into ten genera. These are *Pseudomonas, Xanthomonas, Stenotrophomonas, Burkholderia, Gluconobacter*, *Ralstonia, Comamonas, Brevundimonas, Sphingomonas*, *and Caulobacter*. Migula (1894) described the genus *Pseudomonas* on the basis of morphological characteristics, and later, Stanier et al. (1966) established the taxonomical (physiological and biochemical properties) basis for the identification of species. Winslow et al. (1917) placed Pseudomonads in the family Pseudomonadaceae. Since then, its taxonomy has undergone many changes.

Based on the DNA–DNA hybridization (DDH) and rRNA–DNA hybridization results (Palleroni 1984), the genus was revised, and a subdivision of five groups was implemented. RNA homology group I includes species, such as *Pseudomonas aeruginosa*, *P. fluorescens, P. syringae, P. putida, P. chlororaphis, P. stutzeri, P. alcaligens, P. pseudoalcaligens,* and *P. mendocina*, and are referred to as genus *Pseudomonas* (sensu stricto) subclass *Gamma Proteobacteria* (Palleroni 2008). The rRNA similarity group II, the genus *Burkholderia*, is mainly composed of pathogens except *P. pickettii*. The rRNA similarity group III represents a new genus *Comamonas* and includes five species. The rRNA group IV represents *Gluconobacter* species, and group V represents *Xanthomomonas*. With the initiation of DNA fingerprinting techniques like ARDRA and RFLP as well as 16S rDNA sequencing, several monophyletic groups have been defined and number of species earlier placed in the genus *Pseudomonas* have moved to different genera, but *Pseudomonas* sensu stricto remains robust and as diverse as originally reported by Stanier et al. (1966). The number of species in the genus has increased every year (10 additional species in 2013 and six in 2014 through October) (Gomila et al. 2015). At present, the number of validly published species includes 144 species (including 10 subspecies); these species are present in the List of Prokaryotic Names with Standing in Nomenclature (Parte 2014).

The 16S rDNA gene from *Pseudomonas* spp. contains 1492 nucleotide position, of which 148 are variable and 65 positions of these are within three hypervariable regions. Primers based on these regions have been used to study the diversity of Pseudomonad isolates from different parts of the world (Moore et al. 1996). Remarkable degree of variability has been reported among strains of species that are phylogenetically closely related based on restriction fragment length polymorphism (Ginard et al. 1997; Rainey et al. 1994).

Bacterial diversity is found to decline with the increasing disturbance frequencies. Total bacterial abundance is, however, higher at intermediate and high disturbance frequencies, compared to low and no-disturbance treatments. Increased disturbance frequency also led to changes in community composition, with changes in overall species composition. With increasing disturbance frequency, phylogenetic species variability (an index of community composition) itself became more variable from one sample to another, suggesting a greater role of chance in community composition.

Pseudomonads being one of the most diverse bacterial genera throughout the planet play a key role in soil. Their distribution in soil plays a key role in plant growth promotion and pathogenicity control. Therefore, it is worthwhile to study the *Pseudomonas* diversity in lesser explored area of natural niches.

In this study, we isolated fluorescent *Pseudomonas* from a lesser explored area of Indian Central Himalayan region, examined their comparative genetic diversity and analyzed their ability to colonize rhizosphere of majority of plants and their plant growth promoting efficiency.

## Material and method

### Isolation and enumeration of bacteria

Soil samples at a depth of 1–10 cm (surface soil) and 2–3 ft (deep soil) were collected from three sites (Pinus forest, Quercus forest, and agricultural land) and air dried. One gram of each soil sample was added to 10 ml of 0.1 % (w/v) distilled water. After homogenization, this solution was serially diluted (10^−1^ to 10^−8^) and aliquots were plated in triplicates on (a) King’s B (g/l) proteose peptone—20, K_2_HPO_4_—1, MgSO_4_·7H_2_O—0.4, glycerol—8 ml, agar–20, pH 7 (King et al. 1954). (b) Gould’s media (g/l) sucrose 10.0, glycerol 10.0, casamino acid 5.0, NaHCO_3_ 1.0, MgSO_4_·7H_2_O 1.0, K_2_HPO_4_ 2.3, sodium lauryl sulfate(SLS) 1.2, trimethoprim 20 mg (filter sterilization) add after medium is autoclaved and cooled, pH 7.4–7.6 (Gould et al. 1985). (c) Nutrient agar. (d) Tryptic soya agar. Plates were incubated at 28 °C, for 24 h. The number of colonies for each dilution plated on both media was enumerated and colony forming units (CFUs) were expressed as:

$${\text{Colony forming units }} = \frac{\text{Number of colonies}}{{{\text{Dilution}}\;{\text{factor}}}} \times {\text{ Volume}} .$$

### Screening for siderophore producing isolates

Two hundred and thirty-eight colonies isolated from pine forest, oak forest, and agricultural land soil were inoculated in King’s B broth for mother culture preparation and incubated at 28 °C and 120 rpm for 24 h. Loop full of overnight grown culture was spot inoculated on chrome azurol ‘S’ agar media (CAS media) for screening of siderophore producing isolates containing: (A) chrome azurol ‘S’, (B) 1 mM FeCl_3_·6H_2_O in HCl, and (C) HTMA in nutrient agar (pH 7) (Schwyn and Neilands 1987). These plates were kept in incubator at 28 °C for 48 h. A total of 51 isolates were found positive on CAS media plate for siderophore production.

### Extraction of DNA

Genomic DNA of all the 56 siderophore producing strains was extracted by a modified method of Bazzicalupo and Fani (1995). Extracted genomic DNA was electrophoresed in 0.8 % agarose gel (with ethidium bromide at a final concentration of 0.5 μg/ml) at 80 volts. Gel was then visualized under ultraviolet light using a gel documentation system, Gel Doc Mega (Biosystematica).

### Identification of presumptive *Pseudomonas* isolates by amplification of 16S rDNA using *Pseudomonas* specific primers

*Pseudomonas* specific primers forward primer Ps-for (5′ GGTCTGAGAGGATGATCAGT 3′) and the reverse primer, Ps-rev (5′ TTAGCTCCACCTCGCGGC 3′) were used to amplify a 990-bp region of 16S rRNA gene (Widmer et al. 1998). Genomic DNA of 51 presumptive *Pseudomonas* isolates was used on thermal cycler, Gen Amp PCR System 9700 (Applied Biosystems). A 50 µl of reaction mixture included, 5 µl (5–10 ng) of bacterial DNA as template, 5 µl of 10X buffer for Taq DNA Polymerase (100 mM of TRIS–HCl and 15 mM MgCl_2_), 2.5 µM of each primer, 250 µM of dNTPs, and one unit of Taq DNA polymerase (Bangalore Genei, India). The reaction condition includes an initial denaturation of 5 min at 95 °C, followed by 35 cycles of 1 min at 94 °C, 1 min at 57 °C and 1 min at 72 °C with the final extension of 10 min at 720 °C. Amplified DNA was visualized after electrophoresis using UV gel documentation system GelDocMega (Biosystematica).

### Restriction fragment length polymorphism analysis

Aliquots of purified 16S rRNA *Pseudomonas* specific gene region (990 bp) amplicon were further digested separately with three restriction endonucleases *Alu*I, *Msp*I, and *Rsa*I restriction enzymes. The reaction mixture included 20-μl aliquot of the amplicon as template, 1X enzyme buffer and 1U/rxn of each restriction enzyme, and was digested at 37 °C for 2 h. Digested products were analyzed by agarose gel (2.5 %) electrophoresis. Data for the presence or absence of bands were scored in binary format (0–1). Jaccard similarity coefficient was used by DendroUPGMA method for construction of dendrograms.

### Physicochemical properties

#### Hydrogen cyanide (HCN) production

Production of HCN for selected isolates was assessed on King’s B medium (KB) containing 4.4 g/l of glycine with indicator paper (Whatman soaked in 0.5 % (w/v) picric acid and 2 % (w/v) sodium carbonate), and plates were incubated at 27 °C for 48 h to 72 h. Change in color of the indicator paper from yellow to cream, light brown, dark brown, and brick red indicates HCN production (Alström and Burns 1989).

#### Phosphate solubilization

Pikovskaya’s agar medium was used to check the phosphate solubilization property (Pikovskaya 1948). Mother culture was prepared in King’s B broth and spot inoculated on the Pikovskaya’s agar plates. The plates were kept for incubation at 28 °C for 24 h. A clear zone indicates the phosphate solubilising property of isolates.

#### Production of indoleacetic acid

Indoleacetic acid (IAA) production was detected as described by Brick et al. (1991). Bacterial cultures were grown for 48 h (*Pseudomonas*) on their King’s B media at 36 ± 2 °C. Fully grown cultures were centrifuged at 3000 rpm for 30 min. The supernatant (2 ml) was mixed with two drops of orthophosphoric acid and 4 ml of the Salkowski reagent (50 ml, 35 % of perchloric acid, 1 ml 0.5 M FeCl_3_ solution). The development of pink color indicates IAA production.

## Results

### Isolation and enumeration of bacteria

A total of 238 bacterial strains were isolated from different soils, and the highest numbers of bacteria were reported from the oak forest soils (surface and deep) followed by pine forest soil and agricultural land soil. The results of this study revealed that the maximum amount of total bacterial population, i.e., 44 % was isolated from oak forest soil. It was further analyzed that oak surface soil (OSS) contributed 24.78 %, followed by oak deep soil (ODS) that represented 20.16 % of total oak bacterial count.

On analyzing the contribution of pine forest in total bacterial count, it was found that only 29 % of total bacterial count was from pine forest soil. The data further exhibited that out of 29 %, the pine forest soil (PSS) contributed 16.80 %, followed by pine deep soil (PDS) 13.86 %. The least percentage of total bacterial count (23 %) was reported from agricultural soil ecosystems. The data showed that agricultural surface soil (ASS) contributed 15.54 % of bacterial count, whereas agricultural deep soil (ADS) contributed only 8.82 % of bacterial count.

Reports have shown that the size and structure of microbial populations are affected by soil type and plant species (Sah et al. 2016). Total bacterial count was analyzed for all six soil types and compared for significant differences among them through one way analysis of variance (ANOVA). It was found that the soil bacterial count differed significantly across all six soil types (*F* = 104.690, *df* = 5; *P* < 0.001). We also explored the data further via Tukey’s post hoc multiple comparisons for soil bacterial count between various soil groups (i.e., a pair by pair analysis). Tukey’s post hoc multiple comparisons revealed that there is a statistically significant difference (*P* < 0.05) in bacterial count when PSS is compared with other soil types, i.e., PDS, OSS, ODS, and ADS (Table 1).

**Table 1:** Tukey’s post hoc test multiple comparisons for soil bacterial count

Multiple comparisons						
Dependent variable: soil bacterial count Tukey HSD						
(I) Soil	(J) Soil	Mean Difference (I−J)	Std. error	Sig. (*P* value)	95 % confidence interval	
					Lower bound	Upper bound
Pine surface soil (PSS)	Pine deep	7.00000*	1.79505	0.020^a^	0.9706	13.0294
	Oak surface	−19.00000*	1.79505	0.000^c^	−25.0294	−12.9706
	Oak deep	−8.00000*	1.79505	0.008^b^	−14.0294	−1.9706
	Agriculture surface	3.00000	1.79505	0.573^ns^	−3.0294	9.0294
	Agriculture deep	19.00000*	1.79505	0.000^c^	12.9706	25.0294
Pine deep soil (PDS)	Pine surface	−7.00000*	1.79505	0.020^a^	−13.0294	−0.9706
	Oak surface	−15.00000*	1.79505	0.000^c^	−21.0294	−8.9706
	Oak deep	−26.00000*	1.79505	0.000^c^	−32.0294	−19.9706
	Agriculture surface	−4.00000	1.79505	0.293^ns^	−10.0294	2.0294
	Agriculture deep	12.00000*	1.79505	0.000^c^	5.9706	18.0294
Oak surface soil (OSS)	Pine surface	8.00000*	1.79505	0.008^b^	1.9706	14.0294
	Pine deep	15.00000*	1.79505	0.000^c^	8.9706	21.0294
	Oak deep	−11.00000*	1.79505	0.001^b^	−17.0294	−4.9706
	Agriculture surface	11.00000*	1.79505	0.001^b^	4.9706	17.0294
	Agriculture deep	27.00000*	1.79505	0.000^c^	20.9706	33.0294
Oak deep soil (ODS)	Pine surface	19.00000*	1.79505	0.000^c^	12.9706	32.0294
	Pine deep	26.00000*	1.79505	0.000^c^	19.9706	17.0294
	Oak surface	11.00000*	1.79505	0.001^b^	4.9706	28.0294
	Agriculture surface	22.00000*	1.79505	0.000^c^	15.9706	44.0294
	Agriculture deep	38.00000*	1.79505	0.000^c^	31.9706	25.0294
Agriculture surface soil (ASS)	Pine surface	−3.00000	1.79505	0.573^ns^	−9.0294	3.0294
	Pine deep	4.00000	1.79505	0.293^ns^	−2.0294	10.0294
	Oak surface	−11.00000*	1.79505	0.001^b^	−17.0294	−4.9706
	Oak deep	−22.00000*	1.79505	0.000^c^	−28.0294	−15.9706
	Agriculture deep	16.00000*	1.79505	0.000^c^	9.9706	22.0294
Agriculture deep soil (ADS)	Pine surface	−19.00000*	1.79505	0.000^c^	−25.0294	−12.9706
	Pine deep	−12.00000*	1.79505	0.000^c^	−18.0294	−5.9706
	Oak surface	−27.00000*	1.79505	0.000^c^	−33.0294	−20.9706
	Oak deep	−38.00000*	1.79505	0.000^c^	−44.0294	−31.9706
	Agriculture surface	−16.00000*	1.79505	0.000^c^	−22.0294	−9.9706

*ns* not significant

*Significance difference

^a^Significance at 5 % (*P* value <0.05)

^b^Significance at 1 % (*P* value <0.01)

^c^Significance at 0.1 % (*P* value <0.001)

There was a significant difference when PDS was compared with PSS, OSS, ODS, and ADS with a mean difference of −7.0, −15.0, −26.0, −15.0, and 12.0, respectively, while no significant difference was observed in PDS and ASS and a mean difference of −4.0 was ODS followed by OSS. The highest mean difference of 38.0 was observed between ODS and ADS bacteria count followed by PDS (26.0), ASS (22.0), PSS (19.0), and OSS (−11.0). A mean difference of −3.0 and 4.0 between ASS and PSS/PDS, respectively, and a value of *P* > 0.05 represents that there is no significant difference in their bacterial count. No significant difference is observed between PSS and ASS (*P* > 0.05, mean difference 3.00), and PDS and ASS (*P* > 0.05, mean difference = −4.00).

### Screening for siderophore producing isolates

All the 238 isolates were investigated for siderophore production. Fifty-six isolates representing a 23.52 % of total bacterial population isolated were positive for siderophore production. Of the 56 siderophore producing isolates, 46.42 % of the isolates belong to the oak forest soil (OSS: 32.14 %; ODS: 14.28 %), 14.99 % from pine forest soil (PSS: 17.85 %; PDS: 7.14 %), and 28.56 % from Agriculture soil (ASS: 23.21 %; ADS: 5.35 %).

We also quantified the siderophore producing *Pseudomonas* and analyzed it statistically for all six soil types and compared for significant differences among them through one way analysis of variance (ANOVA). It was observed that the siderophore producing *Pseudomonas* differed significantly across all 6 soil types (*F* = 18.900, *df* = 5; *P* < 0.001). We explored the data further via Tukey’s post hoc multiple comparisons for siderophore producing *Pseudomonas* between various soil groups (i.e. a pair by pair analysis).

Tukey’s post hoc multiple comparisons for siderophore producing *Pseudomonas* reveals significant differences in different types of soil from Uttarakhand State of Indian Himalayan Region (IHR) (Table 2). A statistically significant difference was observed in number of siderophore producing *Pseudomonas* when PSS soil was compared to other soil types, e.g., PDS, OSS, and ADS (*P* < 0.05). A significant difference was also observed in bacterial count of PSS and oak surface soil, while no significant difference was observed between PSS and OSS (*P* > 0.05). When the total number of siderophore producing *Pseudomonas* in PDS was compared with various soil types, e.g., PSS, ODS, and ASS with a mean difference of −7.00, −15.00, −7.00, respectively, it exhibited a statistically significant difference (*P* < 0.05). No statistically significant difference was observed in siderophore producing *Pseudomonas* between PDS and ADS (*P* > 0.05), whereas a significant difference was observed in siderophore producing *Pseudomonas* count when OSS was compared with various soil types, e.g., PSS, PDS, ODS, ASS, and ADS with a mean difference of 8.00, 15.00, 11.00, 8.00, and 15.00, respectively.

**Table 2:** Tukey’s post hoc test multiple comparisons for siderophore producing *Pseudomonas* isolates

Multiple comparisons						
Dependent variable: siderophore producing *Pseudomonas* count Tukey HSD						
(I) Soil	(J) Soil	Mean Difference (I−J)	Std. error	Sig. (*P* value)	95 % confidence interval	
					Lower bound	Upper bound
Pine surface soil (PSS)	Pine deep soil	7.00000	1.82574	0.022^a^	0.8675	13.1325
	Oak surface soil	3.00000	1.82574	0.009^b^	−3.1325	9.1325
	Oak deep soil	−8.00000	1.82574	0.589^ns^	−14.1325	−1.8675
	Agriculture surface soil	0.00000	1.82574	1.00^ns^	−6.1325	6.1325
	Agriculture deep soil	7.00000	1.82574	0.022^a^	0.8675	13.1325
Pine deep soil (PDS)	Pine surface soil	−7.00000	1.82574	0.022^a^	−13.1325	−0.8675
	Oak surface soil	−4.00000	1.82574	0.309^ns^	−10.1325	2.1325
	Oak deep soil	−15.00000	1.82574	0.000^c^	−21.1325	−8.8675
	Agriculture surface soil	−7.00000	1.82574	0.022^a^	−13.1325	−0.8675
	Agriculture deep soil	0.00000	1.82574	1.00^ns^	−6.1325	6.1325
Oak surface soil (OSS)	Pine surface soil	8.00000	1.82574	0.009^b^	1.8675	14.1325
	Pine deep soil	15.00000	1.82574	0.000^c^	8.8675	21.1325
	Oak deep soil	11.00000	1.82574	0.001^b^	4.8675	17.1325
	Agriculture surface soil	8.00000	1.82574	0.009^b^	1.8675	14.1325
	Agriculture deep soil	15.00000	1.82574	0.000^c^	8.8675	21.1325
Oak deep soil (ODS)	Pine surface soil	−3.00000	1.82574	0.589^ns^	−9.1325	3.1325
	Pine deep soil	4.00000	1.82574	0.309^ns^	−2.1325	10.1325
	Oak surface soil	−11.00000	1.82574	0.001^b^	−17.1325	−4.8675
	Agriculture surface soil	−3.00000	1.82574	0.589^ns^	−9.1325	3.1325
	Agriculture deep soil	4.00000	1.82574	0.309^ns^	−2.1325	10.1325
Agriculture surface soil (ASS)	Pine surface soil	0.00000	1.82574	1.00^ns^	−6.1325	6.1325
	Pine deep soil	7.00000	1.82574	0.022^a^	0.8675	13.1325
	Oak surface soil	3.00000	1.82574	0.589^ns^	−3.1325	9.1325
	Oak deep soil	−8.00000	1.82574	0.009^b^	−14.1325	−1.8675
	Agriculture deep soil	7.00000	1.82574	0.022^a^	0.8675	13.1325
Agriculture deep soil (ADS)	Pine surface soil	−7.00000	1.82574	0.022^a^	−13.1325	−0.8675
	Pine deep soil	0.00000	1.82574	1.00^ns^	−6.1325	6.1325
	Oak surface soil	−4.00000	1.82574	0.309^ns^	−10.1325	2.1325
	Oak deep soil	−15.00000	1.82574	0.000^c^	−21.1325	−8.8675
	Agriculture surface soil	−7.00000	1.82574	0.022^a^	−13.1325	−0.8675

*ns* not significant

^a^Significant at 5 % (*P* value <0.05)

^b^Significant at 1 % (*P* value <0.01)

^c^Significant at 0.1 % (*P* value <0.001)

### Identification of presumptive *Pseudomonas* isolates using *Pseudomonas* specific primer

Of the 56 isolates, PCR amplification of 16S rDNA *Pseudomonas* specific region yielded a single amplicon of 990 bp for 51 isolates. Hence, these 51 isolates belong to *Pseudomonas* (sensu stricto) group. The five isolates which were not amplified may not be from within the *Pseudomonas* (sensu stricto) group.

**Fig. 1:**
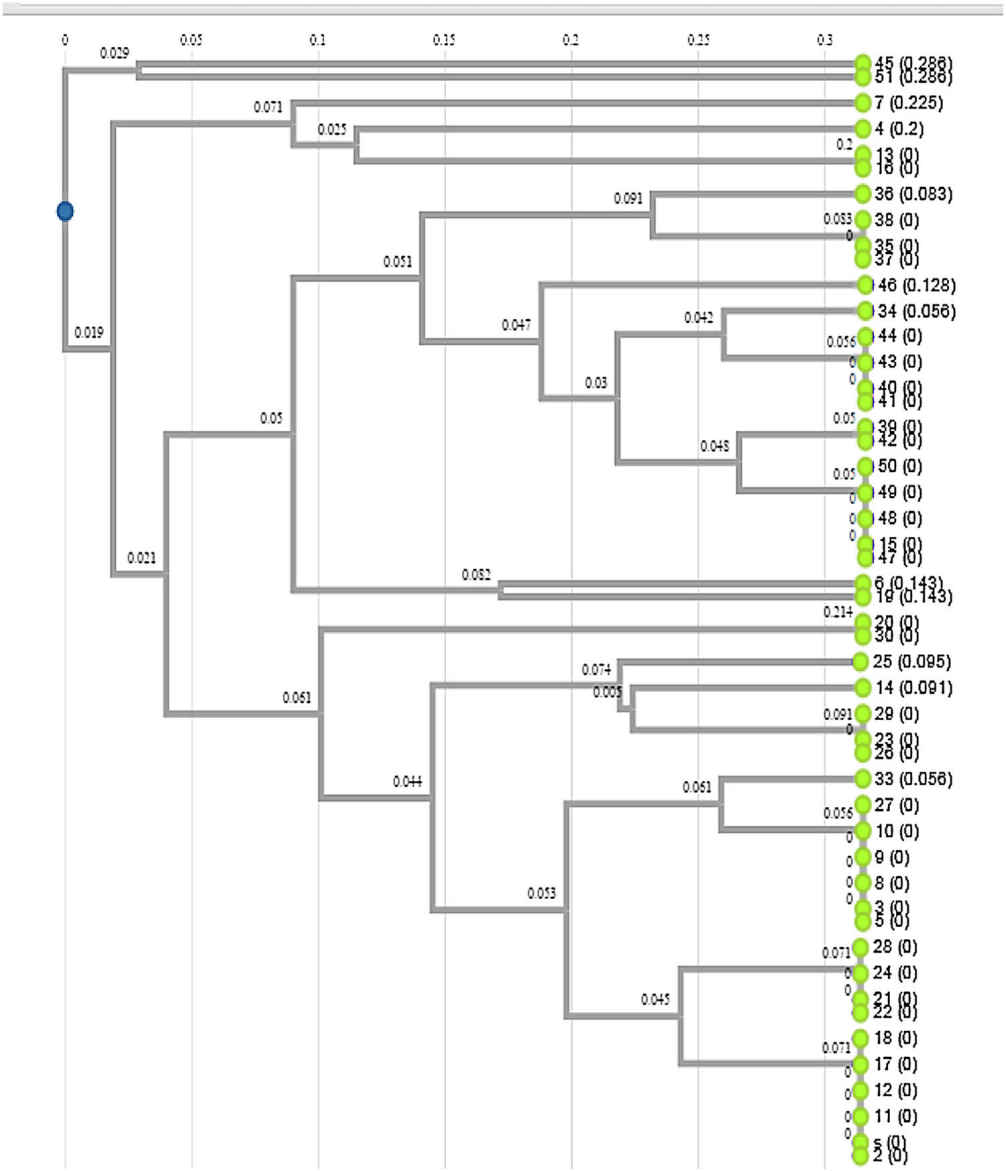
Phylogenetic relationship among *Pseudomonas* isolates from pine forest, oak forest, and agricultural land of Himalayan region

Genetic variation among the isolates was analyzed by RFLP of PCR product with three restriction endonucleases (*Alu*I, *Msp*I, and *Rsa*I). Different profiles having four to seven fragments with variation in size were observed after digestion and dendrogram was constructed by Dendro UPGMA method (Fig. 1). The unique digestion patterns observed segregated the isolates into different groups and subgroups. The representatives from various groups were sequenced for conformation.

The dendrogram represented eight different clusters and six outlying branches based on Jaccard’s similarity coefficient. Isolates showing less than 75 % similarity were considered as outlying branches. The isolates representing >75 to 99 % similarity were considered as one cluster, and isolates representing <75 to 99 % were considered as different cluster or an outlying branch. The branches of dendrogram signify that there is heterogeneity among the isolates

Cluster I included the highest number of isolates. This was split into two, split 1(a) includes four isolates and split 1(b) includes six isolates. The sequencing results revealed the identity of isolates belonging to cluster 1(a) and 1(b) as *P. aeruginosa.* Cluster II, the second largest cluster includes seven isolates. This cluster is split into two groups. 2(a) includes a single isolate, identified as *Pseudomonas. sp.*, while 2(b) includes six isolates. All the isolates in cluster 2(b) are 100 % similar. Clusters I and II share 50 % similarity among them. Cluster III is split into 3(a) and 3(b). Isolates belonging to cluster 3(b) represent 100 % similarity among them. Cluster 3(a) represented by one isolate is 60 % similar to the split 3(b). Cluster III represents 55 % similarity to the outlying branch (OB1) with a single isolate. The second outlying branch (OB2) includes two isolates 20 and 30 representing 100 % similarity among them. Cluster IV includes two isolates representing 50 % similarity among them. Cluster V is split into 5(a) that includes two isolates 39 and 42 having 100 % similarity among them. Isolate 42 is identified as *P. tolassi*, isolate 39 representing 100 % similarity with 42 may also be *P. tolassi.* 5(b) includes isolate 50 identified as *P. fluorescens* and shares 100 % similarity with isolates 15, 47, 48, and 49. Isolates 15, 47, 48, and 49 may also be *P. fluorescens.* Cluster VI is split into two 6 (a) and 6 (b), having 75 % similarity among them. Cluster 6(a) includes a single isolate and cluster 6(b) includes four isolates having 100 % similarity among them. Isolate 46 forms an outlying branch (OB 3). OB3 is less than 60 % similar to Cluster VI. Cluster VII is split into two as 7(a) and 7(b). 7(a) includes a single isolate which is 70 % similar with 7(b). Isolate 35 identified as *P. putida* is 100 % similar with 37 and 38. Hence, it can be said that the isolates belonging to cluster 7(b) may be *P. putida*. Cluster VIII is split into 8(a) and 8(b) with 40 % similarity among them. Isolate belonging to 8(b) represent 100 % similarity among them. Isolate 7 forms an outlying baranch (OB4). Isolates 45 and 51 having less than 15 % similarity among them form two outlying branch OB5 and OB6.

Since the digestion site for the enzymes AluI (AG↓CT) and RsaI (GT↓AC) was different, different restriction patterns were observed. The isolates representing 100 % similarity on dendrogram may be same or with a difference in their subspecies. The sequencing results support the study that the isolates belong to different subgroups of *Pseudomonas* (sensu stricto). The isolates were segregated into different groups as *P. aeruginosa*, *P.* spp, *P. tolassi*, *P. fluorescens*, *P. putida*, and *P. korensis*, representing the heterogeneity among the isolates. Isolates from lesser or undisturbed lands of oak and pine forests were placed in all the clusters as the isolates from agricultural land or a disturbed land. In some of the clusters, isolates from forest and agricultural land are 100 % similar. All of the *Pseudomonas* isolates were further explored for their PGPR properties.

### Physicochemical properties

All the 51 *Pseudomonas* strains were investigated for the plant growth promotory (PGP) traits that include production of siderophore, P-solubilization, HCN production, and IAA production. Results indicate that all the 51 isolates were siderophore producers and IAA producers. It was found 68.62 % isolates were phosphate solubilizers and 62.74 % were HCN producers. It was observed that 50 % (23 strains) of the isolates were positive for all the PGP traits. Out of these 23 isolates 12 belonged to oak forest, 6 to pine forest and 5 to the agricultural land. These 23 isolates belong to different phylogenetic groups formed according to the RFLP results. *Pseudomonas* strains are beneficial and possess PGP traits and hence are explored the most. Among the more important attributes are, the ability to colonize roots successfully (colonization potential), sustain competition through release of bioactive molecules, build up large populations during the growth stage of a plant in soil (Table 3).

**Table 3:** Physicochemical properties of the *Pseudomonas* isolates

Strain	Siderophore production	Phosphate solubilisation	HCN production	IAA production	Strain	Siderophore production	Phosphate solubilisation	HCN production	IAA production
1	+	−	−	+	27	+	+	+	+
2	+	+	+	+	28	+	+	−	+
3	+	+	+	+	29	+	+	+	+
4	+	−	+	+	30	+	+	+	+
5	+	−	+	+	31	+	+	+	+
6	+	+	+	+	32	+	−	−	+
7	+	+	−	+	33	+	−	−	+
8	+	+	+	+	34	+	+	+	+
9	+	−	+	+	35	+	−	−	+
10	+	−	−	+	36	+	+	+	+
11	+	+	+	+	37	+	−	+	+
12	+	+	+	+	38	+	+	−	+
13	+	+	+	+	39	+	−	+	+
14	+	+	+	+	40	+	+	+	+
15	+	−	−	+	41	+	+	+	+
16	+	−	−	+	42	+	+	−	+
17	+	+	−	+	43	+	+	+	+
18	+	+	−	+	44	+	+	−	+
19	+	+	+	+	45	+	−	+	+
20	+	+	+	+	46	+	+	−	+
21	+	+	−	+	47	+	+	+	+
22	+	+	+	+	48	+	+	+	+
23	+	+	+	+	49	+	+	−	+
24	+	+	+	+	50	+	+	−	+
25	+	−	+	+	51	+	−	−	+
26	+	+	+	+					

*HCN* hydrogen cyanide, *IAA* indoleacetic acid production

+ *Pseudomonas* strains showing positive results for the respective test

− *Pseudomonas* strains showing negative results for the respective test

## Discussion

The diversity of plants and animals in forest and agroecosystem has been explored well, whereas the diversity of microorganism is often ignored. The greatest microbial diversity appears to reside in the soil (Zhou et al. 2003). Reduction in soil microbial diversity will result in the reduction of the functional capability of soil (Giller et al. 1997) as soil microorganisms play a very important role in soil fertility not only because of their ability to carry out biochemical transformation but also due to their importance as a source of mineral nutrients, cycling, and availability of nutrients required by the biological systems, the formation of soil organic matter, and decomposition of organic residues and detoxification of soil contaminants (Hendrix et al. 1990; Lavelle 1994) There have been studies on the distribution of microbial diversity in various environmental niches (Stolp 1988). Therefore, the diversity of microorganisms can be explored beneficially.

This study involves *Pseudomonas* from the forests (pine and oak) and agricultural lands of Indian Central Himalayan region. It was observed that higher number of *Pseudomonas* were isolated from pine surface, oak surface, and agricultural surface than the pine deep, oak deep, and agricultural deep soil. The alkalinity of oak soil (Upadhyay et al. 1989) supports the growth of *Pseudomonas* bacteria, while falling pine leaves and pesticide sprays make the pine soil and agricultural soil acidic, respectively, leading to decrease in the presence of *Pseudomonas*. Oak forests are also rich in the nutrients availability and fire free (Champion and Seth 1968) resulting in more microbial community biomass in oak soil.

Out of the 238 isolates, 21.4 % of the total population was identified as siderophore producing *Pseudomonas*. These siderophores producing *Pseudomonas* isolates form eight different clusters that represent the different species or subspecies of *Pseudomonas* sensu stricto. Widmer et al. (1998) identified *P. aeruginosa* and other species, such as *P. fluorescens*, *P. putida*, and *P. syringae*, as members of a phylogenetically homogeneous group *Pseudomonas* (sensu stricto). All the 51 isolates were identified as *Pseudomonas spp., P. aeruginosa*, *P*. *tolassi*, *P. fluorescens*, *P. putida*, and *P. korensis* clustered in different groups. Some of the isolates share 100 % identity among them, irrespective of their place of isolation. Isolates belonging to untouched lands of oak and pine forests were further explored for their physicochemical properties along with isolates from Agricultural land.

Growth promotion and disease control by *Pseudomonas* is complex interrelated processes involving direct and indirect mechanisms that include synthesis of some metabolites, production of siderophore, antibiotics, hydrogen cyanide (HCN), and volatile compounds. Others include mineral solubilization (e.g., phosphorus), competition, and induced systemic resistance (Adesemoye et al. 2008). Siderophore produced by fluorescent *Pseudomonas* rapidly colonizes the roots and significantly increases the plant growth by 144 % in field tests (Kloepper et al. 1980; Sah and Singh 2015). In this study, 56 strains were siderophore producers, and these strains could be efficiently utilized for the plant growth promotion as siderophores mobilize insoluble iron from soil and increase biological availability for plants. Siderophores directly stimulate the biosynthesis of other antimicrobial compounds by increasing the availability of these minerals to bacteria (Sayyed et al. 2005). Phosphorus, an essential element for the plant health is made available to the plants by P-solubilizing microorganisms via production of organic acids. Phosphate-solubilizing fluorescent *Pseudomonas* brings about mobilization of insoluble phosphates in the soil and increase plant growth under conditions of poor phosphorus availability. In this study, we found that 68.62 % of the isolates were able to solubilize phosphate indicating its utilization to provide soluble phosphate to the plant. In another study it was found that, 58.85 % of the isolates were sharp P-solubilizers (Yadav et al. 2014). The secondary metabolite hydrogen cyanide (HCN) produced by fluorescent Pseudomonads is believed to be involved in the suppression of root pathogens. In this study, 62.74 % isolates were found to produce HCN indicating its ability to inhibit root pathogens. Other researchers, e.g., Wani et al. (2007) and Ryall et al. (2009), also reported that HCN producing isolates help in the plant growth. The different *Pseudomonas spp*. (*Pseudomonas entomophila*) produce HCN and effectively suppress soil borne plant diseases. These strains could promote plant growth directly or indirectly or synergistically (Voisard et al. 1989). All of the 51 *Pseudomonas* isolates were able to produce IAA. IAA (indole-3-acetic acid) is the member of the group of phytohormones and is generally considered the most important native Auxin. IAA in different microorganism–plant interactions highlights the fact that bacteria use this phytohormone to interact with plants as part of their colonization strategy, including phytostimulation and circumvention of basal plant defense mechanisms (Voisard et al. 1989).

These tests reveal that 45.01 % of the total isolates were able to show all the PGP traits checked. It was observed that isolates belonging to the undisturbed lands of oak forest were more efficient than isolates from pine forest and agricultural land. A higher number of isolates positive for all the properties (siderophore production, phosphate solubilization, HCN production, IAA production) were isolated from oak forest soil followed by pine forest and agriculture land (oak forest > pine forest > agricultural land). The PGPR properties of the isolates belonging to the unexplored forest soil can be beneficial for enhancing the plant productivity.
